# Pre-eclampsia during pregnancy and risk of endometrial cancer: a systematic review and meta-analysis

**DOI:** 10.1186/s12905-023-02408-x

**Published:** 2023-05-12

**Authors:** H Jordao, K Herink, Eastwood KA, L McVicker, C Kearns, ÚC McMenamin

**Affiliations:** 1 Centre for Public Health, Queen’s University Belfast, Institute of Clinical Sciences-B Building, Royal Victoria Hospital site, Grosvenor Rd, Belfast, Northern Ireland BT12 6BJ UK; 2 Department of St. Michael’s Hospital, Bristol, UK

**Keywords:** Endometrial Cancer, Endometrial neoplasms, Pre-Eclampsia, Pregnancy, Systematic review

## Abstract

**Background:**

Pre-eclampsia may be associated with the development of endometrial cancer; however, previous findings have been conflicting.

**Objectives:**

To investigate if pre-eclampsia is associated with an increased risk of endometrial cancer.

**Method:**

Two independent reviewers screened titles and abstracts of studies identified in MEDLINE, Embase, and Web of Science databases from inception until March 2022. Studies were included if they investigated pre-eclampsia and subsequent risk of endometrial cancer (or precursor lesions). Random-effects meta-analysis was used to calculate pooled hazard ratios (HRs) and 95% confidence intervals (CIs) for the association between pre-eclampsia during pregnancy and endometrial cancer risk.

**Main results:**

There were seven articles identified which investigated endometrial cancer, of which one also investigated endometrial cancer precursors. Overall, the studies include 11,724 endometrial cancer cases. No association was observed between pre-eclampsia and risk of endometrial cancer with moderate heterogeneity observed (pooled HR 1.07, 95% CI 0.79–1.46, I^2^ = 34.1%). In sensitivity analysis investigating risk of endometrial neoplasia (atypical hyperplasia, carcinoma in situ, or cancer), there was some evidence that pre-eclampsia was associated with an increased risk (HR 1.34, 95% CI 1.15–1.57, I^2^ = 29.6%).

**Conclusions:**

Pre-eclampsia was not associated with an increased risk of endometrial cancer. Additional large studies with information on pre-eclampsia sub-type aiming to investigate endometrial cancer precursor conditions are merited.

**Supplementary Information:**

The online version contains supplementary material available at 10.1186/s12905-023-02408-x.

## Introduction

The incidence of endometrial cancer has increased rapidly in high-income countries [1], doubling in the UK over the last 30 years [2] and in the US, a 0.5% increase in age-adjusted rates has been observed year-on-year [3]. Rising incidence has occurred in almost all global regions, likely due to increasing obesity rates, greater life expectancy, and changes in reproductive patterns [4].

Pregnancy is known to confer long-term protection against endometrial cancer, most likely due to pregnancy-induced hormonal changes [5, 6]. The hormonal milieu of pregnancy is characterised by increased levels of estrogen, progesterone, and intrauterine growth factors, almost exclusively produced by the placenta [7]. Pre-eclampsia, a pregnancy-induced syndrome resulting in placental dysfunction [8], complicates 2–7% of pregnancies and rates have been rising over the past two decades [9]. A dominant feature of pre-eclampsia is anti-angiogenesis [10], which restricts tumor growth [11] and pre-eclampsia has therefore been hypothesised to be associated with a reduction in the risk of solid tumors in later life.

There is limited understanding about the relationship between pre-eclampsia and the risk of endometrial cancer despite some evidence linking pre-eclampsia with a reduced risk of breast cancer [12]. Similar to breast cancer, most endometrial cancers are hormone-dependent with primary risk factors related to exposure to endogenous and/or exogenous estrogens [13]. There is some evidence that pre-eclamptic women have lower levels of estrogen and elevated levels of progesterone compared to women with normotensive pregnancies [14, 15]. Reduced levels of insulin-like growth factor-1 (IGF-1), a potent stimulator of endometrial cell proliferation, have been observed in women with pre-eclampsia [16]. In contrast, circulating levels of androgens in women with pre-eclampsia are elevated approximately two-to three-fold [17] with emerging evidence suggesting that androgens may be important in driving endometrial cancer development and progression [18].

Findings from epidemiological studies investigating pre-eclampsia and risk of endometrial cancer have been inconsistent. A large Danish case-control study did not find any association between pre-eclampsia and the risk of endometrial cancer [19]. In contrast, a nested case-control study using registry data from four Nordic countries, including 10,924 endometrial cancer cases, concluded that pre-eclampsia during pregnancy was associated with a significantly elevated risk of endometrial cancer, with similar results for both Type I and Type II endometrial cancer [20].

Further investigation of the long-term impacts of pre-eclampsia may provide insights into understudied biological mechanisms of endometrial carcinogenesis. This study aimed for the first time to systematically review the current evidence on the association between pre-eclampsia diagnosed during pregnancy and risk of endometrial cancer.

## Materials and methods

### Search strategy

This systematic review was carried out and reported according to the Preferred Reporting Items for Systematic Reviews and Meta-analyses (PRISMA) guidelines, see supplementary Tables 2 [21, 22] as well as the Meta-analyses Of Observational Studies in Epidemiology (MOOSE) checklist, see supplementary Table 1 [22]. The protocol for this review is registered on the Prospero database 2020: CRD42020213459 [23].

The search was carried out using three online databases; EMBASE (Reed Elsevier PLC Amsterdam, Netherlands), MEDLINE (US National Library of Medicine, Bethesda, Maryland, USA), and Web of Science (Thompson Reuters, Times Square, New York, USA), from database inception until March 2022. The search strategy contained relevant Medical Subject Heading (MESH) and keywords relating to pre-eclampsia and endometrial cancer (or endometrial precursor conditions), see Table 1. A broad search strategy containing terms related to any cancer type was employed to prevent relevant studies from being missed. Validated study design search filters for observational studies were used [24] and the search was restricted to studies in humans [25]. Abstracts and unpublished studies were excluded. No restrictions on language were applied.

**Table 1:** Search Strategy (Medline)

#	Searches
1	Pre-eclamp*.mp. or Pre-Eclamp*/
2	Preeclamp*.mp.
3	Tox?emia.mp. or Toxemia/
4	Gestosis.mp.
5	Hypertensive pregnancy disorder*.mp.
6	Cancer*.mp. or Neoplasms/
7	Neoplasm*.mp. or Neoplasms/
8	tumo?r*.mp.
9	Malignan*.mp.
10	Carcinoma/ or Carcinoma.mp.
11	Adenocarcinoma/ or Adenocarcinoma*.mp.
12	Adenosarcoma/ or Adenosarcoma.mp.
13	Carcinosarcoma/ or Carcinosarcoma*.mp.
14	Atypical hyperplasia.mp.
15	Hyperplasia with atypia.mp.
16	Intraepithelial neoplasia.mp.
17	Epidemiologic Studies/
18	exp Case-Control Studies/
19	exp Cohort Studies/
20	Case control.tw.
21	(cohort adj (study or studies)).tw.
22	Cohort analyS.tw.
23	(Follow up adj (study or studies)).tw.
24	(observational adj (study or studies)).tw.
25	Longitudinal.tw.
26	Restrospective.tw.
27	Cross sectional.t.w
28	1 or 2 or 3 or 4 or 5
29	6 or 7 or 8 or 9 or 10 or 11 or 12 or 13 or 14 or 15 or 16
30	17 or 18 or 19 or 20 or 21 or 22 or 23 or 24 or 25 or 26 or 27
31	28 and 29 and 30
32	Limit 31 to humans

### Inclusion and exclusion criteria

All titles and abstracts were independently screened, and relevant abstracts had their full texts reviewed independently, by at least two reviewers (ÚCM, KH, KAE, LMV, HJ). Articles which met the following pre-set criteria were eligible for inclusion:

i. **Participants**: Women and girls with a confirmed pregnancy.
ii. **Interventions**: Recorded diagnosis of pre-eclampsia at any stage during pregnancy.
iii. **Comparators**: Women and girls with a confirmed pregnancy without a diagnosis of pre-eclampsia during pregnancy.
iv. **Outcome**: Endometrial cancer was the primary outcome and endometrial precursors (such as atypical endometrial hyperplasia) was a secondary outcome.
v. **Study design**: Observational studies (including case-control, retrospective, and prospective cohorts).

Studies were included if they reported a risk estimate and 95% confidence interval (CI) or if there was sufficient information provided to calculate an estimate. Any discrepancies were resolved through discussion. Bibliographies of included studies were also reviewed. However, studies which included younger patients or did not report pregnancy and endometrial cancer or premalignant endometrial lesions, were excluded from this analysis.

### Data extraction

Relevant information concerning the author, publication year, study location, study design, study population characteristics, information on pre-eclampsia diagnosis, duration of follow-up, confounders, information on endometrial cancer diagnosis, and study results were extracted from the full-text articles. The Newcastle-Ottawa Scale (NOS) was used to derive a quality score for each of the studies included in the review [26].

### Statistical analysis

Risk estimates for the association between pre-eclampsia and endometrial cancer risk, including relative risks (RR), odds ratios (OR), or hazard ratios (HR), and corresponding 95% CIs were extracted from each study. ORs and RRs in this instance should roughly approximate a HR as endometrial cancer is not a common outcome [27, 28]. Multivariate estimates were prioritised for the meta-analysis but if not provided univariate estimates were used.

The risk estimates and associated 95% CIs were converted to log values and a random-effects model [27] was used to statistically pool results using the ‘metan’ package in STATA version 17.0. To assess heterogeneity between studies, the I [2] statistic was calculated [29] with I [2] values of 25%, 50%, and 75% described as low, moderate, and high heterogeneity [29].

A sub-group analysis was carried out restricting to higher quality (NOS score of ≥ 7) and lower quality (NOS score of < 7) studies. A sub-group analysis was also conducted restricted to studies that adjusted for confounding factors (including body mass index, (BMI) and maternal age). Additionally, a sensitivity analysis was conducted to include studies that investigated the risk of any endometrial neoplasia, including precursor conditions (such as endometrial hyperplasia). Finally, to evaluate their individual effect on the pooled estimate, a sensitivity analysis was conducted whereby each study was systematically removed from the main analysis.

## Results

**Fig. 1:**
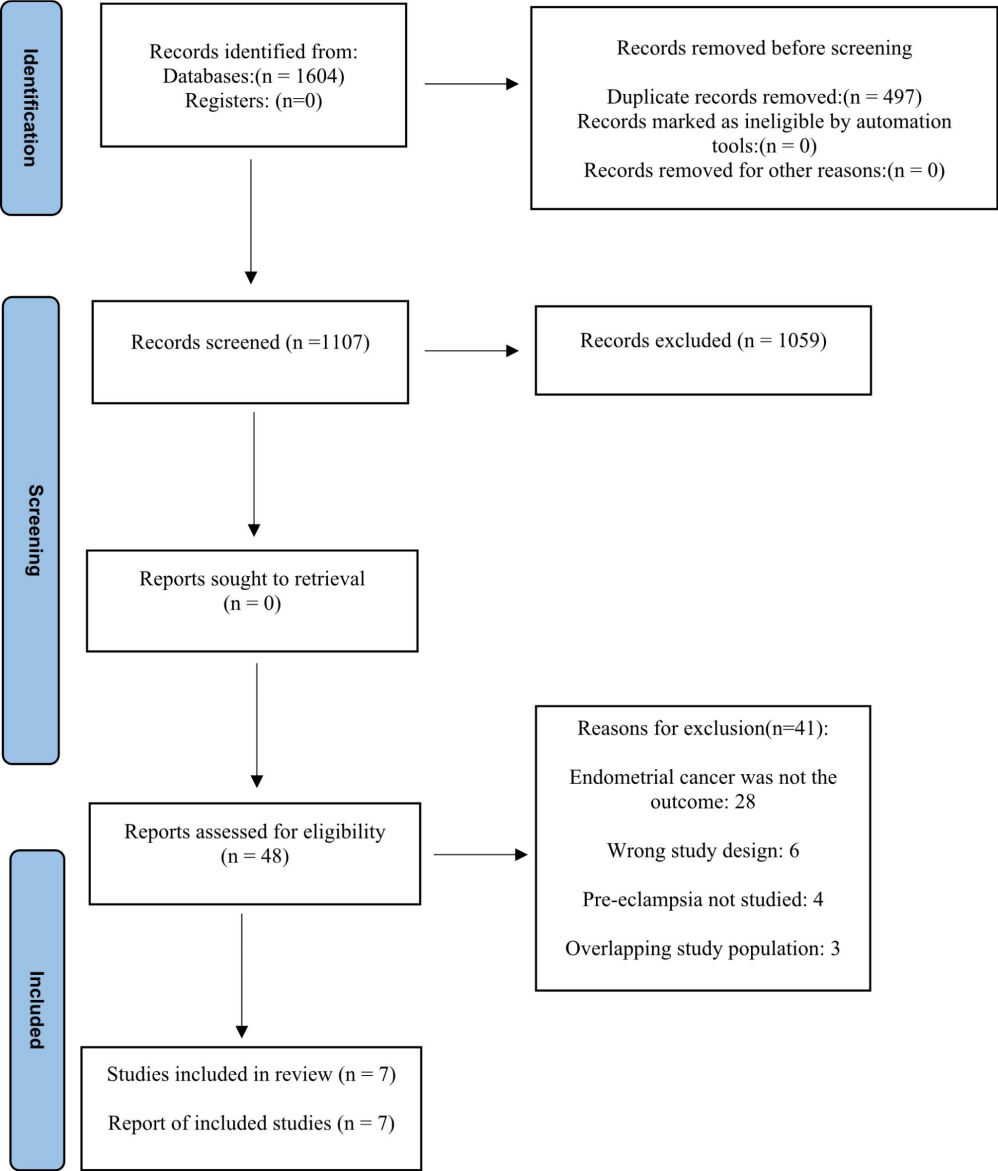
Flow chart of the selection process to identify studies investigating pre-eclampsia and risk of endometrial cancer

A flowchart displaying the study selection process is outlined in Fig. 1. Following the removal of duplicates, 1,107 records were screened by title and abstract. A total of 48 articles were identified for full-text review and of these, seven studies met the inclusion criteria [20, 30–35].

There was some potential overlap in study populations in two of the identified articles [20, 32] however, following personal communication with study authors, we retrieved results from one study which restricted to an earlier time period, therefore eliminating potential participant overlap [32].

### Study characteristics

The characteristics of the included studies are detailed in Table 2. Three studies were conducted in Europe [20, 31, 32] while four were in Asia [30, 33–35]. Five studies were cohort in design, [30–34] one utilised a nested case-control approach [20] and another one was case-control [35]. Five studies were population-based, [20, 31, 33–35] one was single-centre-based [30] and one was multi-centre based [32]. In total, 714,286 women were included and 11,724 endometrial cancer cases were identified, however, sample sizes varied across studies, Table 2.

**Table 2:** Characteristics of studies investigating pre-eclampsia and risk of endometrial cancer

Author (Year) Location	Study Design	Study Cohort/ Database	Study Population	Recruitment Period	Study Size	Pre-eclampsia Ascertainment/	Endometrial Cancer Ascertainment	Pre-eclampsia definition	No. Endometrial Cancer Cases	Follow-up (mean/median)	NOS score	Adjustments
Liu et al.(35) **(2021)** China	Population-based retrospective case-control study	Wuxi Maternity and Child Health Hospital, Nanjing Medical University database	Women diagnosed with endometrial cancer and controls who had more than one live birth	2013–2016	532	Wuxi Maternity and Child Health Hospital database	Medical Records	SP ≥ 140mmHg and/or DP ≥ 90mmHg measured on two occasions separated by at least 6 h, with or without proteinuria, and/or impaired liver function and/or lower platelet count, after 20 weeks of gestation in accordance with the guidelines of the ISSHP.	189	Not stated	5/9	None listed
Trabert et al. (20) **(2020)** Nordic countries Denmark, Finland, Norway, Sweden	Population-based nested case-control study	Denmark, Finland, Norway, and Sweden Nationwide Health Registers	Women with a pregnancy lasting at least 22 weeks	Denmark: 1973–2011, Finland: 1987–2012, Norway: 1967–2013, Sweden: 1974–2013	134,673	National patient and Birth Registries	National Cancer Registry	Not defined.	10,924	46 years (maximum)	9/9	Country, Categorical birth year, Age at index date, Marital status at first birth, pre-pregnancy or early-pregnancy BMI at the last pregnancy, and parity.
Cho et al. (34) **(2019)** South Korea	Retrospective population-based cohort study	Korea National Health Insurance (KNHI) claims database	Women who gave birth in 2007.	2007	386,614	KNHI database	KNHI database	Defined according to ICD-8 codes.	75	8 years (maximum)	7/9	Age at birth, Advanced maternal age, Primiparity, Multifetal pregnancy, C/S, pre-eclampsia, postpartum haemorrhage, placental abruption, placenta previa, and uterine arterial, embolization
Table 2***cont’d***: **Characteristics of studies investigating pre-eclampsia and risk of endometrial cancer (continued)**												
Walfisch et al. (30) **(2015)** Southern Israel	Hospital-based retrospective cohort study	Soroka University Medical Center, Southern Israel	Women who delivered during the study period (1988–2013)	1988–2013	103,180	Peri-natal database	Hospital Records	New onset hypertension with proteinuria during pregnancy.	57	11.6 years (mean)	4/9	None listed
Bhattacharya et al.(31) **(2012)** United Kingdom (Aberdeen)	Retrospective population-based cohort study	The Aberdeen Maternity and Neonatal	Primiparous women born on/before 31st December 1967	1950–2007	25,791	AMND database	Scottish Cancer Registry	Gestational hypertension (DP > 90mmHg on 2 occasions at least 4 h apart or a single reading of 110mmHg; from 20 weeks gestation onwards in a previously normotensive woman) Plus at least 1 episode of proteinuria of 0.3 g/24 h.	194	1,109,329-woman years (maximum)	8/9	Year of birth, smoking status, social class of women at the time of first pregnancy
Calderon-Margalit et al.(33) **(2009)** Western Jerusalem	Retrospective population-based cohort study	Jerusalem Perinatal study	Women with a pregnancy lasting at least 28 weeks	1964–1976	37,927	Birth notifications, Maternity wards logbooks	Israel Cancer Registry	Hypertension (SP > 140 mmHg and/or DP > 90 mmHg) proteinuria, and oedema.	183	33.52 years (mean)	7/9	Age at first birth
Mogren et al. (32) **(2001)** Sweden	Multi-centre retrospective cohort study	Västerbotten and Vasternorrland counties.	Primiparous women	1955–1973	25,569	Local Birth Registry	National Cancer Registry	Defined according to ICD-8 codes.	102	41 years (maximum)	7/9	None listed

EC: Endometrial cancer, DP: Diastolic pressure, SP: Systolic pressure, IRR: Incidence Rate Ratio, HR: Hazard Ratio, OR: Odds Ratio, ISSHP: International Society for the Study of Hypertension in Pregnancy, NOS: Newcastle Ottawa scale

All studies included pregnancies complicated by pre-eclampsia, identified through medical records or national birth registries, Table 2. Definitions of pre-eclampsia varied between studies including (I) new-onset hypertension with proteinuria during pregnancy [30] (II) pre-eclampsia defined according to ICD-8 codes [34] (III) pre-eclampsia considered as a triad of hypertension (SP > 140 mmHg and/or DP > 90 mmHg) proteinuria, and oedema [33]. The included studies evaluated either the absence or presence of pre-eclampsia with no reference to subtypes (i.e., early-onset or late-onset).

Endometrial cancer diagnoses were ascertained from cancer registries in four studies, [20, 31–33] hospital records in two studies [30, 35] and a health insurance database in one study [34]. In terms of study quality, most studies were defined as being of moderate quality, however only four adjusted for potential confounders [20, 31, 33, 34]. Only one study investigated the association between pre-eclampsia and endometrial cancer subtypes (Type I and Type II) [20] and one study provided additional results for a combined outcome of endometrial neoplasia which included atypical endometrial hyperplasia, carcinoma in situ of the endometrium, or endometrial cancer [34].

### Pre-eclampsia and risk of endometrial cancer

**Fig. 2:**
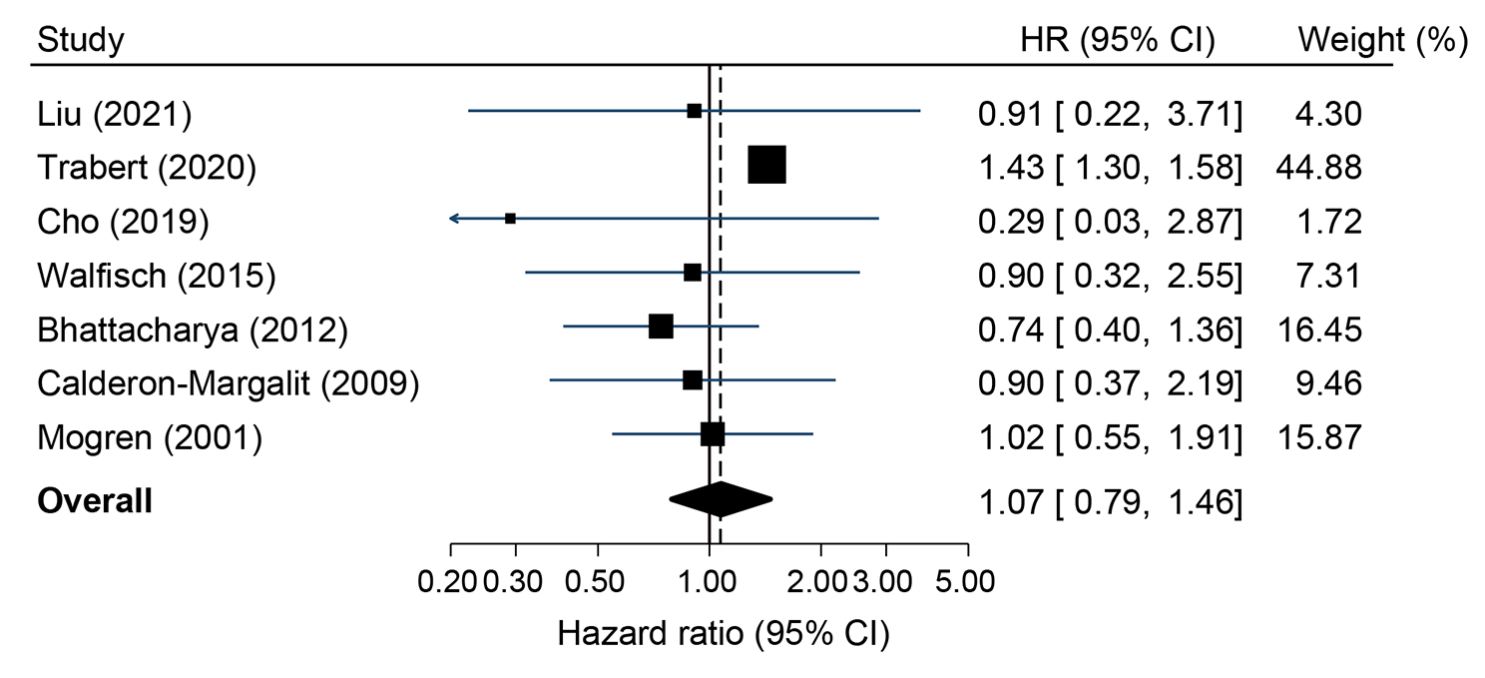
Meta-analysis of studies investigating pre-eclampsia and risk of endometrial cancer

Figure 2 shows the results of the pooled analysis for all studies, showing no significant association between pre-eclampsia and risk of endometrial cancer, with moderate heterogeneity observed (pooled HR 1.07, 95% CI 0.79–1.46, I^2^ = 34.1%).

Results were largely similar in sub-group analysis based on study quality; (NOS score ≥ 7: pooled HR 1.05, 95% CI 0.72–1.54, I^2^ = 50.7%, NOS score < 7: pooled HR 0.90, 95% CI 0.39–2.08, I^2^ = 0.0%), Table 3. When studies were restricted to those that included adjustments for potential confounding factors, results were similar to the main analysis, Table 3. Only one study provided results by endometrial cancer type and found that pre-eclampsia was associated with an increased risk of both Type I (OR 1.44, 95% CI 1.30–1.59) and Type II endometrial cancer (OR 1.39, 95% CI 0.91–2.15), but numbers were reduced in the Type II analysis [20]. A sensitivity analysis evaluating the risk of any sub-type of endometrial neoplasia showed that pre-eclampsia was associated with an increased risk of atypical hyperplasia, carcinoma in situ, or cancer with a pooled HR of 1.34 (95% CI 1.15–1.57) with moderate heterogeneity (I^2^ = 38.5%), Table 3. In additional sensitivity analyses removing individual studies, results were largely similar to the main analysis however, the pooled estimate increased when the study by Bhattacharya et al. [31] was excluded (pooled HR 1.40, 95%CI 1.26–1.54, I^2^ = 0.0%), Table 3.

**Table 3:** Sub-group and sensitivity analyses investigating pre-eclampsia and risk of endometrial cancer

Sub-group analyses	No. of included studies	Pooled risk estimate (95% CI)	I-squared	P-value (Heterogeneity)
**Main analysis**	**7**	**1.07 (0.79–1.46)**	**34.1%**	**0.17**
Studies with a quality score ≥ 7	5	1.05(0.72–1.54)	50.7%	0.08
Studies with a quality score of < 7	2	0.90(0.39–2.08)	0.0%	0.99
Restricted to studies adjusting for potential confounding factors	4	1.01(0.61–1.67)	58.1%	0.06
Risk of endometrial neoplasia^a^	7	1.34(1.15–1.57)	29.6%	0.20
**Summary of pooled results removing individual studies investigating pre-eclampsia and risk of endometrial cancer**				
Excluding Liu et al. (2021)	6	1.06(0.75–1.48)	42.9%	0.12
Excluding Trabert et al. (2020)	6	0.85(0.60–1.21)	0%	0.92
Excluding Cho et al. (2019)	6	1.12(0.84–1.49)	31.7%	0.19
Excluding Walfisch et al. (2015)	6	1.07(0.76–1.50)	40.8%	0.13
Excluding Bhattacharya et al. (2012)	6	1.40(1.26–1.54)	0%	0.42
Excluding Calderon-Margalit et al. (2009	6	1.07(0.76–1.51)	39.1%	0.15
Excluding Mogren et al. (2001)	6	1,04(0.71–1.52)	38.9%	0.15

^a^ Result from Cho et al. (2019) [31] included atypical endometrial hyperplasia, carcinoma in situ of the endometrium or endometrial cancer

## Discussion

### Main findings

This is the first systematic review and meta-analysis to evaluate the association between pre-eclampsia and risk of endometrial cancer. Pooled results showed that pre-eclampsia was not significantly associated with the risk of developing endometrial cancer and results were similar when restricted to higher quality studies.

### Interpretation

Despite the inclusion of 714,286 women and 11,724 EC cases, only seven studies were identified in this systematic review, and the findings varied between individual studies. The study by Trabert et al. [20] conducted within four Nordic countries included the largest number of endometrial cancer cases (n = 10,924) and contributed over 44.9% to the weighting in the meta-analysis. In contrast to the pooled analyses, in this study endometrial cancer risk was significantly increased by 43% for women who had a diagnosis of pre-eclampsia during pregnancy compared to women with a normotensive pregnancy [20]. The increased risk was similar in stratified analysis by Type I and Type II endometrial cancer [20]. Type I endometrial cancers are typically less aggressive and are more estrogen-sensitive [36] compared to Type II endometrial cancers however, recent studies have debated this [37]. Although the Nordic study did not provide information on the specific clinical definitions of pre-eclampsia, it used high-quality national patient registries to capture information on pre-eclampsia and endometrial cancer types [20]. It was also the only study in the review to adjust for BMI; obesity is a risk factor for both pre-eclampsia and endometrial cancer [38]. The findings from the Trabert et al. [20] study contrast with a previous population-based Danish registry study that was excluded from this review due to overlapping study populations with the Nordic study [20]. Hallum et al. [19] found no association between pre-eclampsia and endometrial cancer risk (OR 1.11, 95% CI 0.68–1.81). The study lacked BMI adjustment but did adjust for age at first birth, parity, diabetes, and educational attainment. Interestingly in sub-group analysis by the timing of pre-eclampsia onset, a notable increased risk of endometrial cancer was associated with early-onset pre-eclampsia (OR 2.64, 95% CI 1.29–5.38) but not late-onset pre-eclampsia (OR 0.73, 95% CI 0.38–1.42), although the analysis only contained nine endometrial cancer cases in each group [19]. None of the included studies in this review stratified results according to early or late stage pre-eclampsia, limiting further investigation.

Sensitivity analysis in this review investigated the risk of any endometrial neoplasia (atypical hyperplasia, carcinoma in situ or cancer) by the additional inclusion of 3,370 cases which resulted in a significant 34% increased risk in pooled analysis. However, only one study investigated these additional outcomes. Further investigation of endometrial cancer precursor conditions is required to elucidate if pre-eclampsia may influence earlier stages of endometrial carcinogenesis. This is especially relevant given that endometrial atypical hyperplasia carries a high risk of progressing to endometrial cancer [39].

Potential biological mechanisms linking pre-eclampsia and endometrial cancer are currently understudied. Elevated androgen levels are observed in women with pre-eclampsia [40], possibly due to insufficient enzyme production within the placenta to induce aromatisation of testosterone to estrogen as well as increased inhibin A levels which results in increased androgen production [41]. Several large prospective investigations have found that increased circulating testosterone concentrations, or genetic markers of higher testosterone levels, in women are linked to an increased risk of endometrial cancer [42, 44]. However, it is unclear if androgens are associated with endometrial cancer risk independently of their being precursors to estrogens or if other metabolic pathways affect risk [43]. Recently, a large prospective US study observed increased risks for endometrial cancer in women with the highest circulating levels of adrenal androgens and high levels of estrogens relative to these androgens, suggesting that androgens likely influence endometrial carcinogenesis via estrogen metabolism as adrenal androgens can be aromatized to estrogens [44]. Other proposed mechanisms include immune modulation underlying pre-eclampsia pathophysiology which may contribute to production of inflammatory cytokines and pro-inflammatory T cells; [45, 46] inflammatory markers and mediators, such as CRP, TNF α and VEGFA, have been positively associated with endometrial cancer risk, independent of obesity [47, 48].

In contrast to the main findings from this review, there is suggestive evidence that pre-eclampsia during pregnancy may be associated with a reduced risk of breast cancer [49, 50]. During pregnancy different factors such as reduced levels of estrogens and IGF-1, elevated level of progesterone, androgen, corticotropin-releasing factors can individually or collectively play a crucial role to reduce the breast cancer risk in women [50, 51]. Alternatively, pre-eclampsia may carry other risks that outweigh any potential positive impacts of hormonal fluctuations. Mechanistic studies are required to further elucidate biological mechanisms that may underly potential associations between pre-eclampsia and hormone-sensitive cancers, including endometrial cancer.

### Strengths and limitations

Despite the large number of endometrial cancers cases included in the meta-analysis (> 11,000), only seven studies met the criteria for our systematic review. Most studies in the review identified pre-eclampsia and endometrial cancer from national registers and medical databases which reduced potential recall bias however, misclassification of exposure is still possible and may have attenuated results. Varying definitions for pre-eclampsia were used across the studies which may reflect differences in study time periods. This was notable in the study by Calderon-Margalit et al. [33] whereby oedema was included as a requirement for diagnosis, therefore women with pre-eclampsia who did not present with this particular symptom were possibly misclassified and not included within the final results. In addition, studies did not provide sufficient information to conduct sub-group analysis for endometrial cancer type (Type I or Type II) or onset of pre-eclampsia (early-onset or late-onset). It also wasn’t possible to stratify results based on important clinical factors such as maternal age, BMI and diabetes. The present meta-analysis included only observational studies. It might present challenges because of inherent biases and differences in study designs yet, they provide a tool for helping to understand and quantify sources of variability in results across studies. Finally, all studies were conducted in European or Asian populations which reduced the generalisability of the findings to more ethnically diverse populations.

## Conclusion

Overall, the findings from this systematic review and meta-analysis suggested no association between pre-eclampsia and subsequent risk of endometrial cancer. There was some weak evidence to suggest that pre-eclampsia was associated with an increased risk of any endometrial neoplasia, but studies were limited. To further elucidate the relationship between pre-eclampsia and endometrial cancer risk, future studies are required and should aim to include large prospective cohorts using validated data to investigate pre-eclampsia onset, as well as endometrial cancer type and precursor conditions.

## Electronic supplementary material

Below is the link to the electronic supplementary material.

Additional File Table 1: Meta-analyses Of Observational Studies in Epidemiology (MOOSE) Checklist. Additional File Table 2: Preferred Reporting Items for Systematic Reviews and Meta-Analyses (PRISMA) Checklist

## Data Availability

The datasets used and/or analysed during the current study are available from the corresponding author on reasonable request.
