# Supplementary material for: Pre-eclampsia during pregnancy and risk of endometrial cancer: a systematic review and meta-analysis
Source: BMC Womens Health. 2023 May 12;23:259. doi: 10.1186/s12905-023-02408-x (PMC10182685; doi:10.1186/s12905-023-02408-x)
Supplement: Supplementary file 1 — Additional File Table 1: Meta-analyses Of Observational Studies in Epidemiology (MOOSE) Checklist. Additional File Table 2: Preferred Reporting Items for Systematic Reviews and Meta-Analyses (PRISMA) Checklist [file 12905_2023_2408_MOESM1_ESM.docx]

**Supplementary table 1: MOOSE (Meta-analyses Of Observational Studies in Epidemiology) Checklist**

| **Section/ topic** | **Item** | **Checklist item** | **Location** |
| --- | --- | --- | --- |
| **Title** | **1** | Identify the report as a systematic review. | **1** |
| **Abstract** | **2** | Abstracts. | **2** |
| **Introduction** | | | |
| **Rationale** | **3** | Describe the rationale for the review in the context of existing knowledge. | **3** |
| **Objectives** | **4** | Provide an explicit statement of the objective or question the review addresses. | **4** |
| **Method** | | | |
| **Eligibility criteria** | **5** | Specify the inclusion and exclusion criteria for the review and how studies were grouped for the syntheses. | **5,6** |
| **Information sources** | **6** | Specify all databases, registers, organisations, reference lists and other sources searched/ consulted to identify studies. Specify the date when each source was last searched or consulted. | **5** |
| **Search strategy** | **7** | Present the full search strategies for all databases, registers, and websites, including any filters and limits used. | **5** |
| **Selection process** | **8** | Specify the methods used to decide whether a study met the inclusion criteria of the review, including how many reviewers screened each record and each report retrieved, whether they worked independently, and if applicable, details of automation tools used in the process. | **5,6** |
| **Data collection process** | **9** | Specify the methods used to collect data from reports, including how many reviewers collected data from each report, whether they worked independently, any processes for obtaining or confirming data from study investigators, and if applicable, details of automation tools used in the process. | **5,6** |
| **Data items** | **10a** | List and define all outcomes for which data were sought. Specify whether all results that were compatible with each outcome domain in each study were sought, and if not, the methods used to decide which results to collect. | **6** |
|  | **10b** | List and define all other variables for which data were sought (participant and intervention characteristics, funding sources). Describe any assumptions made about any missing or unclear information. | **6** |
| **Study risk of bias assessment** | **11** | Specify the methods used to assess risk of bias in the included studies, including details of the tool(s) used, how many reviewers assessed each study and whether they worked independently, and if applicable, details of automation tools used in the process. | **5,6** |
| **Effect measures** | **12** | Specify for each outcome the effect measure (RR, mean difference) used in the synthesis or presentation of results. | **6** |
| **Synthesis methods** | **13a** | Describe the processes used to decide which studies were eligible for each synthesis (tabulating the study intervention characteristics and comparing against the planned groups for each synthesis) | **5,6** |
|  | **13b** | Describe any methods required to prepare the data for presentation or synthesis, such as handling of missing summary statistics, or data conversions. | **5** |
|  | **13c** | Describe any methods used to tabulate or visually display results of individual studies and syntheses. | **6** |
|  | **13d** | Describe any methods used to synthesise results and provide a rationale for the choice. If meta-analysis was performed, describe the model, method to identify the presence and extent of statistical heterogeneity, and software package used. | **6,7** |
|  | **13e** | Describe any methods used to explore possible causes of heterogeneity among study results (subgroup analysis). | **6** |
|  | **13f** | Describe any sensitivity analyses conducted to assess robustness of the synthesised results. | **6,7** |
| **Reporting bias assessment** | **14** | Describe any methods used to assess risk of bias due to missing results in a synthesis (arising from reporting biases). | **7** |
| **Certainty assessment** | **15** | Describe any methods used to assess certainty (or confidence) in the body of evidence for an outcome. | **7** |
| **Results** | | | |
| **Study selection** | **16a** | Describe the results of the search and selection process, from the number of records identified in the search to the number of studies included in the review, ideally using a flow diagram. | **8** |
|  | **16b** | Cite studies that might appear to meet the inclusion criteria, but which were excluded, and explain exclusion reason. | **8** |
| **Study characteristics** | **17** | Cite each included study and present its characteristics. | **10** |
| **Risk of bias in studies** | **18** | Present assessments of risk of bias for each included study. | **10, Fig2** |
| **Results of individual studies** | **19** | For all outcomes, present, for each study: (a) summary statistics for each group (where appropriate) and (b) an effect estimates and its precision (confidence/credible interval), ideally using structured tables or plots | **Table 2 and 3** |
| **Results of syntheses** | **20a** | For each synthesis, briefly summarise the characteristics and risk of bias among contributing studies. | **10, 11**  **Figure 2** |
|  | **20b** | Present results of all statistical syntheses conducted. If meta-analysis was done, present for each the summary estimate and its precision (confidence/credible interval) and measures of statistical heterogeneity. | **Figure 2** |
|  | **20c** | Present results of all investigations of possible causes of heterogeneity among study results. | **10,11**  **Table 3** |
|  | **20d** | Present results of all sensitivity analyses conducted to assess the robustness of the synthesised results | **Table 3** |
| **Reporting biases** | **21** | Present assessments of risk of bias due to missing results (arising from reporting biases) for each synthesis assessed | **Table 3** |
| **Certainty of evidence** | **22** | Present assessments of certainty (or confidence) in the body of evidence for each outcome assessed. | **Fig 2** |
| **Discussion** | | | |
| **Discussion** | **23a** | Provide a general interpretation of the results in the context of other evidence | **13** |
|  | **23b** | Discuss any limitations of the evidence included in the review | **15** |
|  | **23c** | Discuss any limitations of the review processes used. | **15, 16** |
|  | **23d** | Discuss implications of the results for practice, policy, and future research. | **15,16** |
| **Other information** | | | |
| **Registration and protocol** | **24a** | Provide registration information for the review, including register name and registration number. | **5** |
|  | **24b** | Indicate where the review protocol can be accessed, or state that a protocol was not prepared | **5** |
|  | **24c** | Describe and explain any amendments to information provided at registration or in the protocol. | **5** |
| **Support** | **25** | Describe sources of financial or non-financial support for the review, and the role of the funders in the review | **Title page** |
| **Competing interests** | **26** | Declare any competing interests of review authors | **17** |
| **Availability of data, code, and other materials** | **27** | Report which of the following are publicly available and where they can be found template data collection forms; data extracted from included studies; data used for all analyses; any other materials used in the review. | Tables and figures |

Page MJ, McKenzie JE, Bossuyt PM, Boutron I, Hoffmann TC, Mulrow CD, et al. The PRISMA 2020 statement: an updated guideline for reporting systematic reviews. BMJ. 2021.

**Supplementary table 2: PRISMA Checklist**

| **Section/topic** |  | **Checklist item** | **Reported on page** |
| --- | --- | --- | --- |
| **TITLE** | | |  |
| Title | 1 | Identify the report as a systematic review, meta-analysis, or both. | 1 |
| **ABSTRACT** | | |  |
| Structured summary | 2 | Provide a structured summary including, as applicable: background; objectives; data sources; study eligibility criteria, participants, and interventions; study appraisal and synthesis methods; results; limitations; conclusions and implications of key findings; systematic review registration number. | 2 |
| **INTRODUCTION** | | |  |
| Rationale | 3 | Describe the rationale for the review in the context of what is already known. | 3 |
| Objectives | 4 | Provide an explicit statement of questions being addressed with reference to participants, interventions, comparisons, outcomes, and study design (PICOS). | 4 |
| **METHODS** | | |  |
| Protocol and registration | 5 | Indicate if a review protocol exists, if and where it can be accessed (e.g., Web address), and, if available, provide registration information including registration number. | 5 |
| Eligibility criteria | 6 | Specify study characteristics (e.g., PICOS, length of follow-up) and report characteristics (e.g., years considered, language, publication status) used as criteria for eligibility, giving rationale. | 5,6 |
| Information sources | 7 | Describe all information sources (e.g., databases with dates of coverage, contact with study authors to identify additional studies) in the search and date last searched. | 5 |
| Search | 8 | Present full electronic search strategy for at least one database, including any limits used, such that it could be repeated. | 5 and Table 2 |
| Study selection | 9 | State the process for selecting studies (i.e., screening, eligibility, included in systematic review, and, if applicable, included in the meta-analysis). | 5,6 |
| Data collection process | 10 | Describe method of data extraction from reports (e.g., piloted forms, independently, in duplicate) and any processes for obtaining and confirming data from investigators. | 6,7 |
| Data items | 11 | List and define all variables for which data were sought (e.g., PICOS, funding sources) and any assumptions and simplifications made. | 5,6 |
| Risk of bias in individual studies | 12 | Describe methods used for assessing risk of bias of individual studies (including specification of whether this was done at the study or outcome level), and how this information is to be used in any data synthesis. | 6 |
| Summary measures | 13 | State the principal summary measures (e.g., risk ratio, difference in means). | 6 |
| Synthesis of results | 14 | Describe the methods of handling data and combining results of studies, if done, including measures of consistency (e.g., I^2^) for each meta-analysis. | 6,7 |
